# A Case of Cerebral Sinus Venous Thrombosis Resulting in Mortality in Severe Preeclamptic Pregnant Woman

**DOI:** 10.1155/2013/402601

**Published:** 2013-02-24

**Authors:** Hatice Ender Soydinc, Ali Ozler, Mehmet Sıddık Evsen, Muhammet Erdal Sak, Abdulkadir Turgut, Serdar Basaranoglu, Abdurrahim Dusak, Mehmet Guli Cetincakmak

**Affiliations:** ^1^Department of Gynecology and Obstetrics, Dicle University School of Medicine, 21280 Diyarbakir, Turkey; ^2^Department of Radiology, Dicle University School of Medicine, 21280 Diyarbakir, Turkey

## Abstract

Cerebral venous sinus thrombosis (CVST) is a rarely encountered condition during pregnancy. A 21-year-old pregnant woman with labour pains was hospitalized in our clinic. Diagnosis of severe preeclampsia was made based on her clinical and laboratory findings. She suffered from convulsive episodes during postpartum period which lead to initiation of treatment for eclampsia. However neurological and radiological examinations were performed after emergence of additional neurological symptoms disclosed the diagnosis of CVST. In this paper, we aimed to present a case with CVST which diagnosis was confused with eclampsia and resulting in maternal mortality.

## 1. Introduction 

Cerebral venous sinus thrombosis (CVST) is a rarely seen condition. Though its actual incidence is not exactly known, it is estimated to be 5/1.000.000 [1]. Among its important etiological factors, pregnancy, puerperium, oral contraceptive (OCS) use, coagulopathies, intracranial infections, cranial tumors, lumbar punction, malignancy, dehydration, inflammatory bowel disease, connective tissue disorders, Behcet's disease, parenteral infusions, and various drugs can be implicated. In 30% of the patients, the etiology cannot be determined [2]. Signs and symptoms of the disease consist of headache, focal, and generalized convulsions, uni- and bilateral paresis, and papilledema [3]. Early diagnosis and treatment in CVST which is potentially fatal are quiet important. However, eclampsia is a severe obstetrical pathology and life-threatening complication of pregnancy, be recognized by the occurrence of tonic-clonic seizures, usually in a patient who has developed preeclampsia. Its incidence is 0.04% in developed countries, while it is 0.1% in developing countries [4]. Headache, visual disturbance, and right upper abdominal quadrant pain are alarming symptoms predicting development of eclamptic seizures. Eclampsia and CVST can be confused with each other especially when they develop at the background of preeclampsia. 

In this paper, we aimed to present a case of CVST which ended up with maternal mortality and was misdiagnosed as eclampsia. 

## 2. Case Presentation

**Figure 1:**
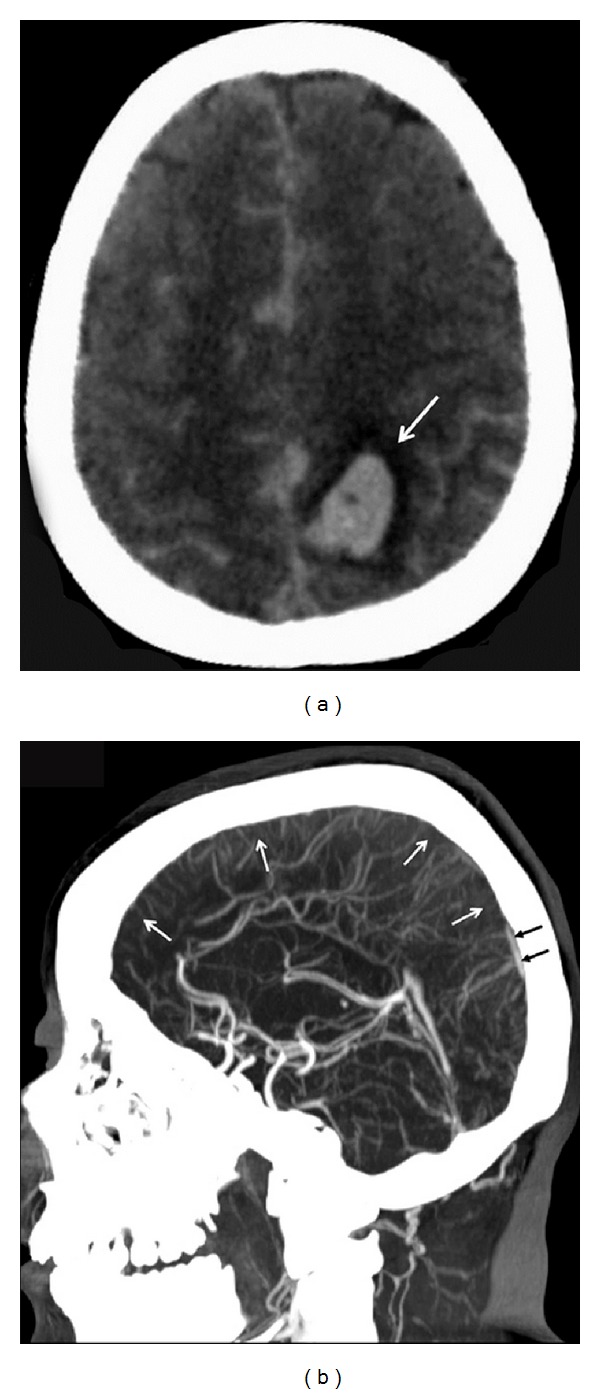
(a) Hemorrhagic area (arrow) is observed on the edematous background at bilateral frontal and parietal regions on noncontrast CT. (b) On sagittal reformat CT angiograms, a thrombus completely filling superior sagittal sinus (white arrows) is observed. Partially confluent sinus (black arrows) and transverse sinus surface patterns are seen.

A 21-year-old pregnant woman, gravida 1, para 0, was hospitalized in our clinic because of her labour pains. On her routine pregnancy controls, any problem and history of epilepsy were not detected. At presentation, her blood pressure (170/100 mm Hg), heart rate (88 bpm), and body temperature (36.1°C) were recorded. On gynecological examination, cervical opening was 8 cm, and fetal head was at +2 station. Sonographic examination detected fetus in 34 weeks of gestation. Her laboratory findings were as follows: aspartate transaminase 100 U/L; alanine transaminase 80 U/L; lactate dehydrogenase 450 U/L; hematocrit 33%; platelets 100,000/mm^3^; and also 3+ proteinuria was in spot urine sample. Hematological and urinary parameters were consistent with severe preeclamptic criteria. The patient gave birth to a male baby weighing 3620 g via vaginally. Apgar scores were 9 and 10 at 1–5 minutes. She suffered from tonic-clonic convulsions and severe headache within the first postnatal hour which was compatible with the diagnosis of eclampsia. Antihypertensives (*α*-methyldopa 250 mg 4 × 2, doxazosin 2 mg 1 × 1) and MgSO_4_ were started. During followup, cranial computerized tomogram (CT) were obtained upon development of somnolence, headache, unilaterally positive Babinski reflexes, and weakness of the left upper and lower extremity muscles. On noncontrast CT, hemorrhagic foci on an edematous background in favour of hemorrhagic infarct on bilaterally frontal and parietal regions were observed that made a mass effect especially on the left posterior parietal region (Figure 1(a)). The patient was monitored in the intensive care unit, and antiedematous (20% mannitol infusion), anticonvulsive (MgSO_4_ and phenytoin capsule 100 mg t.i.d.), and antihypertensive (alpha-methyldopa 4 × 2 and nimodipine tablet 30 mg 6 × 1) treatments were initiated. Detection of a hemorrhagic component presented a dilemma as for initiation of anticoagulant therapy. 

CT angiographic examination was performed on the patient whose neurological symptoms deteriorated and became somnolent. Her CT angiograms demonstrated a thrombosed superior sagittal sinus (Figure 1(b)). Diagnosis of hemorrhagic infarct secondary to venous thrombosis was made, and as an anticoagulant therapy i.v. heparin was planned under strict monitorization of INR (international normalized ratio) values. However clinical state of the patient deteriorated rapidly before initiation of anticoagulant therapy, she became unconscious, and cardiac arrest developed. Despite cardiopulmonary resuscitation, she did not demonstrate any improvement and died. Nearly 5 hours passed between the onset of postpartum convulsions and her death.

## 3. Discussion

 Cerebral venous sinus thrombosis (CVST) is a rarely seen entity which presents diagnostic difficulties, because of the variable nature of its clinical signs and symptoms. Frequently, larger sinuses like superior sagittal sinus are affected. Patients present mostly with complaints of headache [5]. Based on the location of the thrombus, the patients may demonstrate focal neurological symptoms including hemiparesis, aphasia, sensorial loss, vertigo, dizziness, seizures, and loss of consciousness. Convulsions are seen in 40% of the cases, and 50% of them are of focal type. Sometimes life-threatening and generalized tonic-clonic convulsions can be seen [6]. Loss of consciousness is a sign of poor prognosis which is associated with higher mortality rates [7]. Our patient presented with severe headache and convulsion during her postpartum period. Her signs mainly suggested diagnosis of eclampsia because the patient had hypertension and proteinuria. Despite antihypertensive and MgSO_4_ treatment, both of her headaches did not ameliorate, and she had additional neurological symptoms. Therefore we suspected a superimposed cranial morbidity. 

 CVST is more frequently encountered in women, and many causative factors have been proposed. However, more often use of oral contraceptive agents (54.3%), hereditary hypercoagulable states (22.4%), and pregnancy-postpartum periods (20.1%) have been implicated as etiological factors [8]. In our patient “pregnancy-postpartum period” was detected as an important risk factor. 

 Radiological examination plays an important role in the diagnosis of CSVT. Although noncontrast CT does not generally demonstrate any abnormal signs in cases without any neurological deficits, it is helpful in cases with observed neurological symptoms. Hyperdense (>70 HU) images of thrombosed cortical veins and dural sinuses can be observed (Figure 1(a)). On MRI, loss of signal-void appearance is the main finding in cases with CVST. CT angiogram can be helpful in differential diagnosis. CVST is more often seen in superior sagittal sinus, left and right transverse sinuses, respectively. In our case, firstly CT was performed upon occurrence of neurological symptoms. Antiedematous, antihypertensive, and anticonvulsive treatments were started after observance of edema, hemorrhagic areas, and signs of hemorrhagic infarcts. Because of deterioration of clinical picture and worsening of somnolent state despite therapy, CT venograms were obtained for differential diagnosis which demonstrated a thrombus in the sagittal sinus (Figure 1(b)). Within a short-time coma, cardiac arrest developed, and the patient was lost before anticoagulant therapy.

 In the acute treatment of CSVT, heparin, and in the maintenance therapy, warfarin are recommended. Available evidence suggests that systemic or localized thrombolysis reduces rates of mortality and morbidity [9, 10]. In our case, because of the marked hemorrhagic component at baseline and subsequent dramatic deterioration of the clinical picture, the patient died without initiation of anticoagulant therapy.

Even though early diagnosis, and treatment hold an important place in the prognosis of CSVT, as clinical manifestation, altered consciousness, papilledema, its acute onset, intracranial bleeding, presence of focal neurological findings, occlusion of internal cerebral vein and sinus rectus indicate a worse prognosis. The majority (85%) of the cases are discharged without any neurological sequelae, and mortality rates range between 2.5 and 20% [10]. Fatal course of our case was associated with the presence of worse prognostic factors as loss of consciousness and focal neurological symptoms. 

 As a result, CVST can present with tonic-clonic convulsions during pregnancy. This condition can be especially confused with eclampsia in patients with symptomatic severe preeclampsia. In case of development of additional neurological symptoms, CVST should be kept in mind together with other intracranial pathologies. The patient should be urgently evaluated with a multidisciplinary approach and should be instituted treatment including anticoagulant drugs without delay.
